# Low-Molecular Weight Polyethylenimine Modified with Pluronic 123 and RGD- or Chimeric RGD-NLS Peptide: Characteristics and Transfection Efficacy of Their Complexes with Plasmid DNA

**DOI:** 10.3390/molecules21050655

**Published:** 2016-05-18

**Authors:** Jing Hu, Wenfang Zhao, Kehai Liu, Qian Yu, Yuan Mao, Zeyu Lu, Yaguang Zhang, Manman Zhu

**Affiliations:** Department of Biopharmaceutics, College of Food Science and Technology, Shanghai Ocean University, Shanghai 201306, China; 15821787592@163.com (J.H.); wfzhao0504@163.com (W.Z.); 15001970365@163.com (Q.Y.); 13262975153@163.com (Y.M.); m18701881332@163.com (Z.L.); edwarddo@163.com (Y.Z.); zhu_manman7526@163.com (M.Z.)

**Keywords:** polycations, RGD, nuclear localization signal, physico-chemical properties, transfection efficiency

## Abstract

To solve the problem of transfection efficiency *vs.* cytotoxicity and tumor-targeting ability when polyethylenimine (PEI) was used as a nonviral gene delivery vector, new degradable PEI polymers were synthesized via cross-linking low-molecular-weight PEI with Pluronic P123 and then further coupled with a targeting peptide R4 (RGD) and a bifunctional R11 (RGD-NLS), which were termed as P123-PEI-R4 and P123-PEI-R11, respectively. Agarose gel electrophoresis showed that both P123-PEI-R4 and P123-PEI-R11 efficaciously condense plasmid DNA at a polymer-to-pDNA *w*/*w* ratio of 3.0 and 0.4, respectively. The polyplexes were stable in the presence of serum and could protect plasmid DNA against DNaseI. They had uniform spherical nanoparticles with appropriate sizes around 100–280 nm and zeta-potentials about +40 mV. Furthermore, *in vitro* experiments showed that these polyplexes had lower cytotoxicity at any concentration compared with PEI 25 kDa, thus giving promise to high transfection efficiency as compared with another P123-PEI derivate conjugated with trifunctional peptide RGD-TAT-NLS (P123-PEI-R18). More importantly, compared with the other polymers, P123-PEI-R11 showed the highest transfection efficiency with relatively lower cytotoxicity at any concentration, indicating that the new synthetic polymer P123-PEI-R11 could be used as a safe and efficient gene deliver vector.

## 1. Introduction

Cancer is one of the most debilitating human diseases and the second cause of death worldwide. Current gene therapy represents a promising alternative for the treatment of cancer. One of the most challenging issues in cancer gene therapy is the shortage of safe and efficient vectors that can specifically deliver therapeutic DNA to the target site with minimum toxicity [1]. Gene delivery vectors can be classified as viral and non-viral kinds. In recent years, non-viral gene vector have been developed rapidly for their non-immunogenicity, easiness of large-scale manufacturing and tailor-made features for specific therapeutic application [2]. Cationic polymers have been frequently studied because they can be efficiently complexed with negatively charged DNA, thereby increasing DNA stability [3]. Among the polycations, polyethylenimine (PEI) as a commercially available material has become one of the most promising and widely studied gene carriers, largely due to its proton-sponge mechanism that can ensure DNA escape from the endocytic pathway efficiently [4,5]. Studies have demonstrated that the gene transfection efficiency and cytotoxicity of PEI are highly related to their chain length and topology. High-molecular-weight (HMW) PEI has high gene transfection efficiency but exhibits pronounced cytotoxicity and can induce membrane damage in the initial stages of treatment [6]. Low-molecular-weight (LMW) PEI exhibits low cytotoxicity, but with low transfection efficiency. To achieve high transfection efficiency as well as low cytotoxicity simultaneously, PEI could be modified by cross linking and like strategies [7,8].

Pluronic block copolymers, which are amphiphilic molecules used as structural elements of the polycation gene-delivery systems, consist of hydrophilic ethylene oxide (EO) and hydrophobic propylene oxide (PO) blocks arranged in a basic A-B-A structure (EO_x_-PO_y_-EO_x_) [9]. Hydrophobic poly-PO chains are reported to offer good pluronic interactions, DNA transport and transgene expression. In contrast, hydrophilic EO chains have the ability to prevent binding of other polymers with the membranes. The systems are stabilized in dispersion by the EO corona in a manner similar to regular pluronic micelles. Pluronic P123 with formula EO_20_-PO_70_-EO_20_, which has a proper hydrophile lipophilic balance (HLB) value, can be degraded to decrease cytotoxicity after cell uptake. Studies [10] showed that although the PEI derivate P123-PEI synthesized by cross-linking LMW-PEI with P123 had good degradability and low cytotoxicity, the transfection efficiency of the polymeric gene vector is not high enough.

The process of DNA complexes delivery should be able to target specific cells, internalize complexes and be taken up into the nucleus. The αvβ3 receptor is known to be highly expressed on tumor cells and tumor angiogenic blood vessels, but rarely detectable on quiescent blood vessels. The highly selective expression of αvβ3 integrin in the neovascular tissue and various tumors allows for its use as a suitable target. Knowing that arginine-glycine-aspartate (RGD) peptide is a key binding moiety that has been shown to bind specifically to αvβ3 integrin receptors [11], we used a linear RGD peptide as a new peptide named R4 in the present study.

The nuclear localization signal (NLS, with the sequence Lys-Lys-Lys-Arg-Lys) is a necessary signal sequence that mediates proteins entering cell nuclei through the nuclear pore complex [12]. NLS has been suggested as a means to promote nuclear delivery of expression constructs [13]. Therefore, we used RGD peptide conjunction with NLS to yield a new chimeric peptide RGD-NLS (named R11), and peptide R4 and bifunctional peptide R11 to modify PEI derivates, respectively, in order to improve cell selection, promote cargo transport and enhance transfection efficiency.

In this study, a polymer matrix was synthesized by cross-linking LMW PEI with P123. The bifunctional peptide R11 was conjugated to P123-PEI to form a new polymeric polycation (P123-PEI-R11). Another polycation P123-PEI-R4 was prepared in the same way. We investigated the physicochemical characteristics and efficiency of these polymeric polycations *in vitro* and compared them with another polycation modified by R18 (short for RGD-TAT-NLS, with the sequence RGD-RKKRRQRRR-KKKRK, which was ever reported) [14]. The purpose of the study was to find an ideal tumor target peptide that could reduce cytotoxicity of polymer matrix P123-PEI, improve its tumor targeting, increase cellular uptake of genes and enhance the therapeutic effect of gene therapy.

## 2. Results and Discussion

### 2.1. Synthesis and Characterization of P123-PEI-R4 and P123-PEI-R11

**Figure 1:**
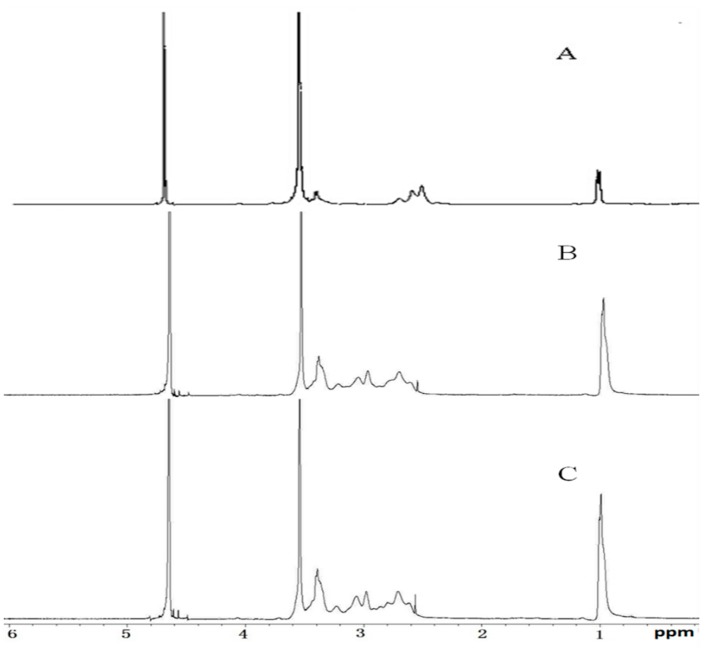
^1^H-NMR spectra of (**A**) P123-PEI; (**B**) P123-PEI-R4; and (**C**) P123-PEI-R11 in deuterium oxide at room temperature.

Figure 1 shows the formation of the new delivery vector P123-PEI, P123-PEI-R4, P123-PEI-R11. These polymers were confirmed by ^1^H-NMR spectroscopy. Figure 1A indicates the ^1^H-NMR spectra of P123-PEI. –CH_2_CH_2_NH– and –CH_2_CH_2_O– proton peaks appeared at δ 2.51–2.7 ppm and δ 3.55 ppm, respectively. The proton peaks of P123-PEI-R4 and P123-PEI-R11 moved towards the lower magnet field when compared with P123-PEI. Meanwhile the –CH_2_CH_2_NH– proton peaks were not found at δ 2.51–2.7 ppm. These changes confirmed that P123-PEI-R4 and P123-PEI-R11 had been synthesized successfully.

### 2.2. Buffer Capacity of Synthesized Polymers P123-PEI-R4, P123-PEI-R11 and P123-PEI-R18

**Figure 2:**
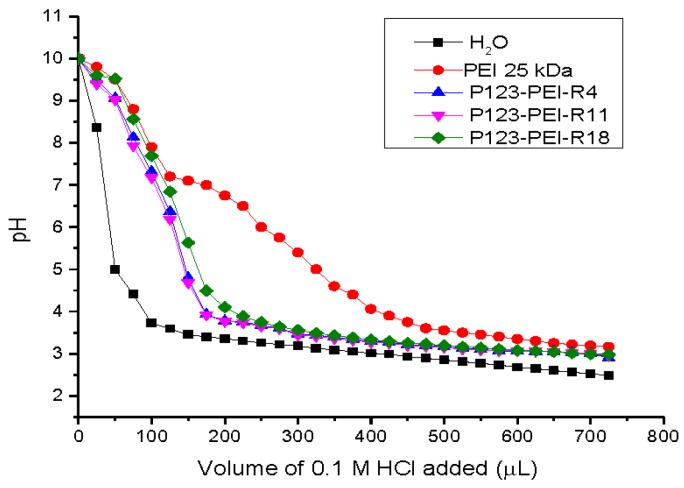
Determination of the buffering capacity of PEI 25 kDa, P123-PEI-R4, P123-PEI-R11 and P123-PEI-R18 by acid-base titration. Each polymer (6 mg) was first dissolved in 30 mL water, and subsequently set to pH 10.0 using 0.1 M NaOH. Then 25 µL increments of 0.1 M HCl were added, and the pH of the solution was measured with a pH meter after each addition. The solutions were titrated to about pH 3.0 with 0.1 M HCI. The titration curve of water is presented as a control.

It is important for gene vector to have well degradation so that to make sure polymers can be degraded and secreted to the extracellular environment to ensure lower cytotoxicity on cells. Cationic polymers have high buffering capacity, which is known as the “proton sponge” effect. This effect may lead to the disruption of endosome in the transfection process, facilitating the escape of polymer/DNA complexes [15,16]. In Figure 2, the acid-base titration curves shows that the branched PEI 25 kDa had a better buffering capacity as compared with derivative PEI. Compared with pure water, P123-PEI-R4, P123-PEI-R11 and P123-PEI-R18 had relatively high buffering capacities in the pH range from 3.5 to 8, indicating that these polymers could be used as potential gene vectors. In addition, as the type of peptide has a mild effect on the buffering capacity, the polymer cross linked with R18 had a slightly higher pH buffering ability than the others.

### 2.3. Particle Size, Zeta Potential Measurements and Morphological Characteristics

**Figure 3:**
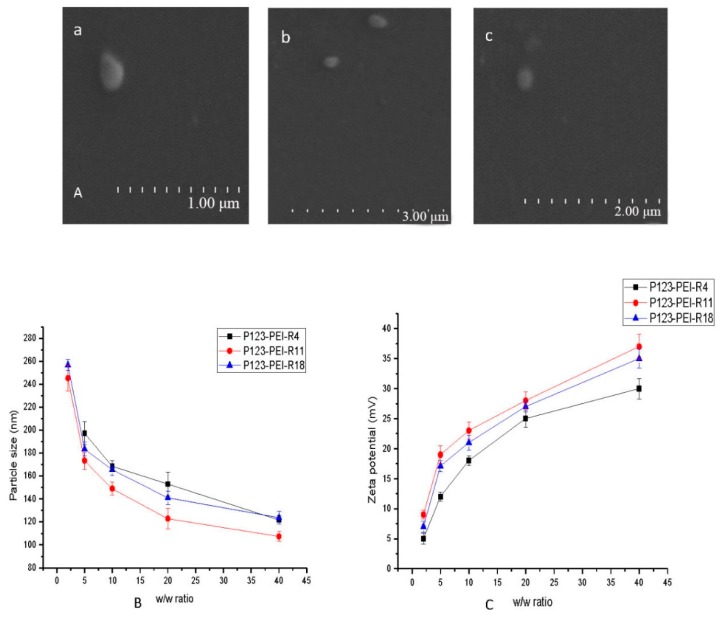
Representative TEM images of P123-PEI-R4/DNA (**a**), P123-PEI-R11/DNA (**b**) and P123-PEI-R18/DNA (**c**); (**B**) Particle size of P123-PEI-R4/DNA, P123-PEI-R11/DNA and P123-PEI-R18/DNA at various polymer-to-pDNA *w*/*w* ratios; (**C**) Zeta potential (mV) of P123-PEI-R4/DNA, P123-PEI-R11/DNA and P123-PEI-R18/DNA at various polymer-to-pDNA *w*/*w* ratios.

Nanoscale particles resulting from DNA condensation by P123-PEI-R4 and P123-PEI-R11 were observed by TEM. As shown in Figure 3A, the polymers P123-PEI-R4/DNA and P123-PEI-R11/DNA had a regular spherical shape at the *w*/*w* radio of 5 and 2, respectively.

Knowing that the size of nanoparticles is important for their substantial effect on the pharmacokinetics and pharmacodynamics [17], we examined the particle size and zeta potential of P123-PEI-R4/DNA, P123-PEI-R11/DNA and P123-PEI-R18/DNA at different *w*/*w* ratios. As shown in Figure 3B, the particle size decreased with the polymer/DNA weight ratio increasing, and tended to be constant with a mean size of 100–280 nm, which is suitable for efficient gene delivery *in vivo*. It was reported that complexes can avoid devouring by macrophage cells and protect humoral DNA within this range of particle size [18]. Meanwhile, at the same weight ratio, P123-PEI-R11/DNA showed a smaller particle size than the others. In addition, no precipitation was observed at any *w*/*w* ratio in the range of the concentrations studied.

With respect to the surface charge, it is important for the surfaces of the complexes to be positively charged to bind to negatively charged cellular membranes and taken up via endocytosis. However, too strong cationic charges will lead to high cytotoxicity.

Figure 3C, the zeta potential of all complexes increased gradually with the charge ratio increasing. In addition, P123-PEI-R11 had a higher zeta potential as compared with the other two polymers at any prepared ratio, probably due to its cross linking with the polymer.

### 2.4. Condensation Status of Plasmid DNA by P123-PEI-R4 and P123-PEI-R11

**Figure 4:**
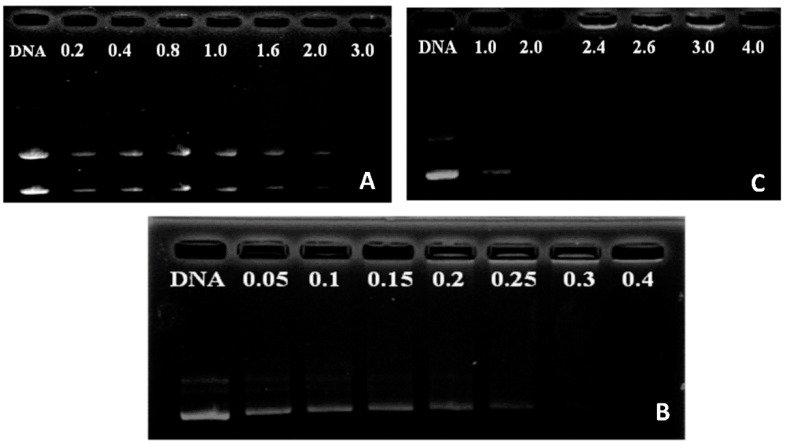
Agarose gel electrophoresis of the complexes at various polymer-to-pDNA *w*/*w* ratios: (**A**) P123-PEI-4/DNA; (**B**) P123-PEI-11/DNA; (**C**) P123-PEI-18/DNA).

Knowing that the cationic property of polymers facilitates their electrostatic interaction with negatively charged nucleic acids and prepared complexes can reduce the electrostatic repulsion between DNA and the cell surface and protect DNA against enzymatic degradation by nucleases in cytoplasm or serum. We assessed the DNA condensation capacity of P123-PEI-R4 and P123-PEI-R11 by observing the electrophoretic mobility of DNA band in agarose gel at various *w*/*w* ratios. As shown in Figure 4, the movement of the plasmid DNA in the gel was retarded and blurry as the amount of the polymer increased, indicating that the polymer bound to the DNA and neutralized its charge. When the *w*/*w* ratio of the polymer and DNA exceeded the neutralization composition, the complex showed a positive charge and stopped migrating toward the anode. As shown in Figure 4B, P123-PEI-R11 was able to effectively condense DNA and neutralize its charge at a *w*/*w* ratio of 0.4, while P123-PEI-R4 and P123-PEI-R18 showed a lower DNA binding ability and could condense DNA at the ratio of 3.0 and 2.0, respectively. These results show that P123-PEI-R11 has the highest DNA binding ability.

### 2.5. Stability of Polymer/pDNA Complexes

**Figure 5:**
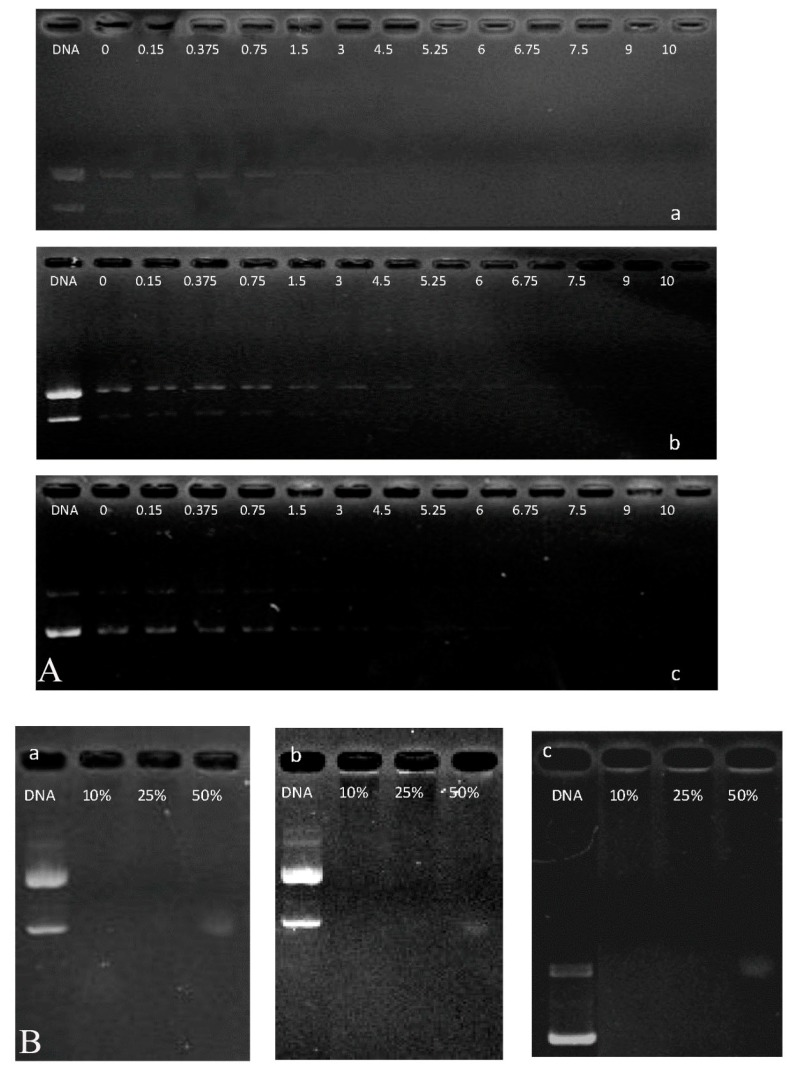
(**A**) Plasmid DNA protection by P123-PEI-R4 (**a**), P123-PEI-R11 (**b**) and P123-PEI-R18 (**c**) from degradation by DNaseI at varying concentrations; (**B**) Plasmid DNA protection by P123-PEI-R4 (**a**), P123-PEI-R11 (**b**) and P123-PEI-R18 (**c**) from degradation by serum at varying concentrations.

Knowing that DNaseI is an endonuclease that catalyzes the hydrolytic cleavage of phosphodiester linkages in the DNA backbone. Considering the abundant amounts of DNaseI in tissue and blood, DNA degradation by DNaseI is a barrier for gene delivery *in vitro* and *in vivo* [19]. We investigated the ability of P123-PEI-R4 and P123-PEI-R11 in protecting DNA from being slaked by DNaseI. The results are shown in Figure 5. By analyzing the further spoliation competition assay, it is obvious that polymer P123-PEI-R4 could protect plasmid DNA from being digested by DNaseI even at the concentration of 3 U DNaseI/µg DNA. P123-PEI-R11/DNA incubated under the same condition exhibited a higher ability to protect plasmid DNA against digestion by DNaseI until at the concentration of 9 U DNaseI/µg DNA. In fact, under the same experimental condition, DNA could be completely digested by DNaseI at a concentration of 0.08 U DNaseI/µg DNA, indicating that both complexes have good DNaseI tolerance.

The stability evaluation of the P123-PEI-R4/DNA and P123-PEI-R11/DNA complex *in vivo* was simulated by treating the complex with serum *in vitro*. As shown in Figure 5B, serum could not dissociate these complexes even at a concentration of 50%, indicating that both complexes may remain stable in blood circulation.

### 2.6. Cytotoxicity Assay

**Figure 6:**
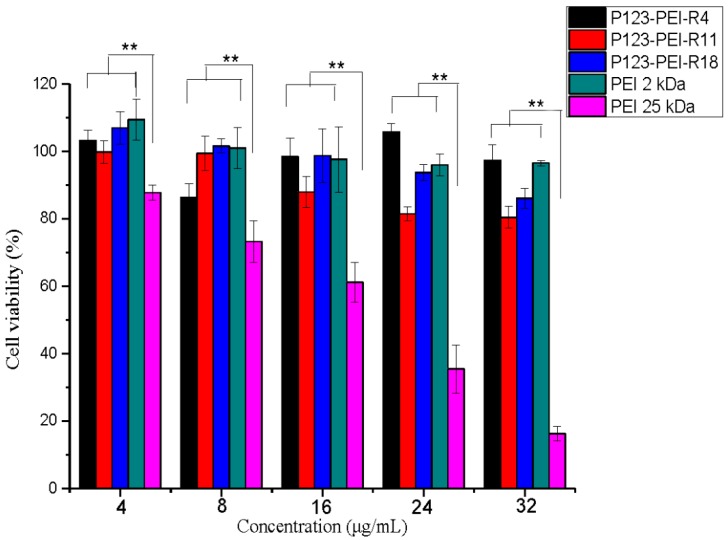
Cytotoxicity of PEI 25 kDa, P123-PEI, P123-PEI-R4, P123-PEI-R11 and P123-PEI-R18 at various concentrations in B16 cell lines using the MTT assay. ** *p <* 0.01.

The positive charge of PEI can cause toxicity to cell. PEI is rich in positive charge owing to the amine groups in the polymer chain. HMW polymer contains more amino groups than LMW polymer which results to higher cytotoxicity [20]. As a result, HMW PEI was limited in transfection due to its high cytotoxicity. In this study, we analyzed the cytotoxicity of P123-PEI-R4 and P123-PEI-R11 in B16 cells by MTT, using P123-PEI, P123-PEI-R18 and PEI 25 kDa as controls. As shown in Figure 6, the cell vitality decreased gradually with the polymer concentration increasing, indicating that a higher polymer concentration would lead to higher cytotoxicity. In addition, P123-PEI-R4, P123-PEI-R11 and P123-PEI-R18 all showed lower cell toxicity than PEI 25 kDa, with the similar cell viability to P123-PEI at any concentration, suggesting that different peptides have little influence on P123-PEI and these polymers have better biocompatibility and the potential to be used as transgenic vectors. The lower cytotoxicity may be due to the lower amino-group density and lower toxic building blocks. As far as P123-PEI-peptide is concerned, the ester bonds of the polymer can degrade into poloxamer oligomers and LMW PEI under physiological conditions, which can be rapidly excluded from cells, thus showing little cytotoxicity.

### 2.7. Transfection Efficiency in Vitro

**Figure 7:**
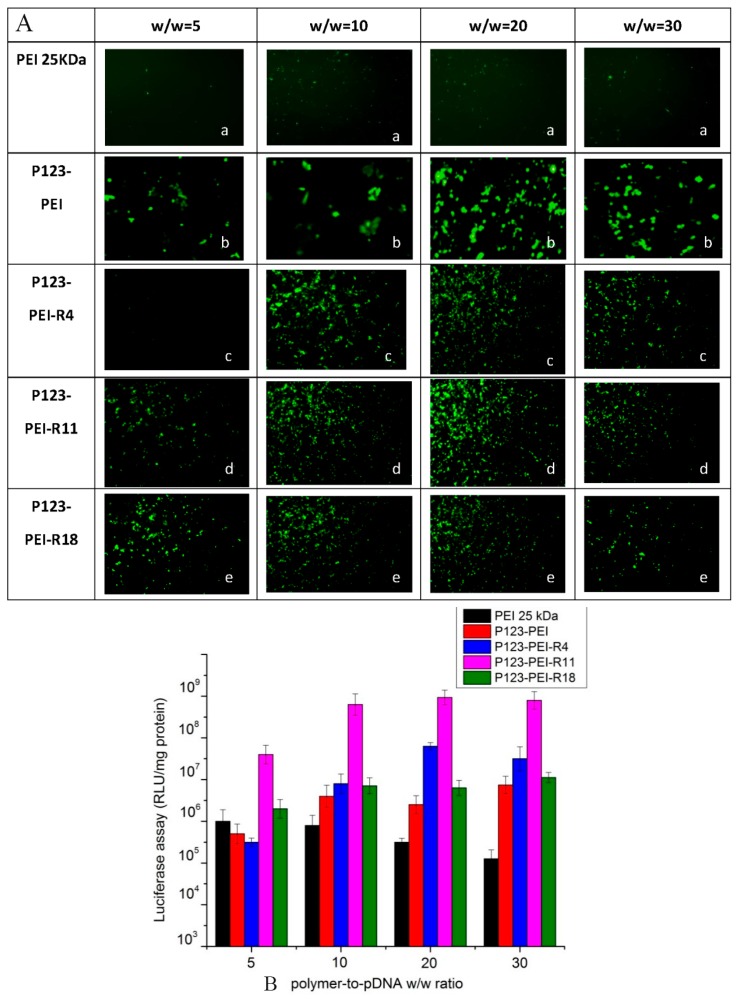
(**A**) Green fluorescent protein (GFP) reporter gene transfection in B16 cells by PEI 25 kDa (**a**); P123-PEI (**b**); P123-PEI-R4 (**c**); P123-PEI-R11 (**d**) and P123-PEI-R18 (**e**) in 24-well plate. Note: 100 µL polymer/DNA complex solution containing 2.5 µg plasmid pEGFP-N2 at various weight ratios ranging from 5 to 30 were added to each well; (**B**) Transfection efficiency of PEI 25 kDa/DNA, P123-PEI/DNA, P123-PEI-R4/DNA, P123-PEI-R11/DNA and P123-PEI-R18/DNA in B16 cells at the polymer/pDNA *w*/*w* ratio of 5, 10, 20, and 30 in the 24-well plate. Note: 100 µL polymer/DNA complex solution containing 2.5 µg plasmid pGL3-Control at various weight ratios ranging from 5 to 30 was added to each well.

As shown in Figure 7A, the polymer/DNA complex modified with different peptides showed different fluorescence strengths at the same dose of DNA (2.5 µg DNA per well).

The EGFP reporter gene expression in B16 cells showed that the derivative PEI had better transfection efficiency than PEI 25 kDa at any weight. Compared with P123-PEI, the complexes crosslinked with peptide showed more fluorescent transfection, indicating that polypeptide helps promote cell transfection. P123-PEI-R11 compounds exhibited higher ability to gene transfer contrast to other complexes at any ratio and also showed the optimal transfection efficiency at the weight ratio of 20. This means that peptide R11 has a better ability to promote the transfection efficiency to complex as compared with the other peptides. These results indicate that the synthetic complexes could serve as an efficient non-viral gene vectors *in vitro*.

Figure 7B showed the result of the gene tranfection efficiency of the four P123-PEI-peptide/pGL3-control complexes at the same dose of DNA (2.5 µg DNA per well) in B16 cells. Transfection efficiency of these complexes showed that the gene vector coupled with peptides has higher transfection efficiency than P123-PEI and PEI 25 kDa. It means that a derivative polymer is necessary, and the functional peptide could enhance transfection efficiency of the complexes *in vitro*.

Compared with P123-PEI-R4, transfection efficiency of P123-PEI-R11 was higher at the designated polymer-to-pDNA *w*/*w* ratios, indicating that NLS has strong influence on transfection of the complexes *in vitro*. The gene vector could translocate DNA into the nucleus after cell uptake and then express it there. Therefore, high efficiency of the complex entering the nucleus might lead to high transfection efficiency of the gene vector/DNA complex. The result of the present study indicated that NLS could enhance the efficiency of the complex entering the nucleus.

As the Figure 7B showed, the transfection efficiency of P123-PEI-R18 was the lowest compared with the other vectors of different peptides, indicating that multiple peptides might weaken the function of an individual peptide. In the Figure 7B showed that transfection efficiency of P123-PEI-R11 was the highest, indicating that RGD-NLS is the most effective of the four peptide-modified P123-PEI.

## 3. Materials and Methods

### 3.1. Reagents and Plasmid DNA

PEI 2 kDa, *N*-succinimidyl-4-(*N*-maleimido-methyl) cyclohexane-1-carboxylate (SMCC) and 3-(4,5-dimethylthiazol-2-yl)-2,5-diphenyl tetrazolium bromide (MTT) were purchased from Sigma-Aldrich (St Louis, MI, USA). P123 was provided by the Second Military Medical University (Shanghai, China). RPMI 1640 medium and fetal bovine serum (FBS) were purchased from Invitrogen (Carlsbad, CA, USA). The peptide Arg-Gly-Asp (R4, MW449.48) and Arg-Gly-Asp-Lys-Lys-Lys-Arg-Lys (R11, MW1314.62) were synthesized by GL Biochem (Shanghai, China). The plasmids of *Escherichia coli* DH5α were amplified by the Tiangen End-Free Mega Plasmid Kit (Hilden, Germany). Benzene, triphosgene, dichloromethane, *N*-hydroxysuccinimide, triethylamine, ethyl acetate, and absolute ethyl alcohol were bought from Sinopharm Chemical Reagent Co., Ltd (Shanghai, China).

### 3.2. Synthesis and Characterization of Polymers

#### 3.2.1. Activation of P123

Before initiation of the experiments, toluene and dichloromethane were dehydrated by co-evaporation. First, the P123 (0.0680 g, 0.0118 mmol) was dried by co-evaporation with toluene under vacuum at 40 °C twice, dissolved in toluene/dichloromethane mixture (3:1, 40 mL), and treated with the double mole of triphosgene (0.0235 mmol) for overnight. Next, the same mole of *N*-hydroxysuccinimide as triphosgene and plenty of triethylamine was added dropwise into benzene/dichloromethane (2:1, 30 mL) after evaporating the primary solvent. After 4-h stirring, the solution was filtered and evaporated to dryness. Finally, 50 mL ethyl acetate was added and centrifuged at 8000 rpm for 15 min. The supernatant was evaporated and the residue was collected.

#### 3.2.2. Synthesis of P123-PEI

Activated P123 (0.01 mmol) was dissolved into 10 mL of anhydrous ethanol as Solution A, and 10-fold mole dehydrated PEI 2 kDa (0.10 mmol) was dissolved in 20 mL anhydrous dichloromethane as Solution B. The molar ratio of PEI with P123 was 10:1. These two reagents were added into 10 mL dichloromethane simultaneously and stirred constantly overnight at room temperature. Then the solution was dialyzed against distilled water at 4 °C for 2 days and lyophilized.

#### 3.2.3. Linking R4 and R11 with P123-PEI

Polymer P123-PEI was conjugated with peptides using SMCC as crosslinker [15]. The SMCC solution (using dimethyl sulfoxide as solvent to 3.33 mg/mL) was added dropwise to P123-PEI solution (using 0.1 M PBS as solvent to 10 mg/mL) at a molar ratio of 2:1 with half an hour stirring at room temperature with gentle shaking. The excessive non-conjugated SMCC was removed by gel chromatography (Sephadex G-25, Pharmacia, Milton Keynes, UK). Then, peptide R4 (using 0.1 M PBS as solvent to10 mg/mL) was mixed into pretreated P123-PEI at a molar ratio of 2:1 with stirring at 4 °C overnight in the dark. The solution was lyophilized after ultrafiltration. The cationic polymer was named P123-PEI-R4. Polymer P123-PEI-R11 was prepared in the same way. ^1^H-NMR spectral analysis was carried out at room temperature after P123-PEI-R4 and P123-PEI-R11 were dissolved in deuterium oxide.

### 3.3. Buffering Capacity of the P123-PEI-R4 and P123-PEI-R11 Polymers

The synthesized polymer solution P123-PEI-R4 and P123-PEI-R11 were prepared in 50 mL flasks (0.2 mg/mL, 30 mL), respectively, using pure water as control. After adjusting the initial pH to 10.0 with 0.1 M NaOH, 25µL increment of 0.1 M HCl were added, and the pH of the solution was measured with a pH meter after each addition.

### 3.4. P123-PEI-R4/DNA and P123-PEI-R11/DNA Complexe Formation

Different charge ratios (*w*/*w*) of P123-PEI-R4/DNA complex were prepared as the ratio of the weight of P123-PEI-R4 and DNA. The ratio of P123-PEI-R11/DNA complex was expressed in the same way. An appropriate polymer solution (PBS 0.1 mol/L, pH 7.4) was added into the aqueous solution with a fixed amount of plasmid DNA (50 ng/µL) with the equal volumes. Then the complexes were incubated at room temperature for 30 min.

### 3.5. Particle Size and Zeta Potential Measurement and Morphologic Observation

Particle size and zeta potential of the polymer/DNA complexes were measured using an electrophoretic light-scattering spectrophotometer (Zetasizer Nano ZS90, MAN0317 Issue 5.0, Malvern Instruments Ltd., Malvern, UK), with a 90° scattering angle in PBS buffer at room temperature. The complexes were prepared at desired *w*/*w* ratios ranging from 0 to 40, and then incubated at 37 °C for 30 min, and measured for size and zeta potential. All the experiments were performed in triplicate in order to ensure the accuracy of the results.

Morphology of the polyplexes was observed by transmission electron microscopy (JEM 2100F, JEOL Ltd., Tokyo, Japan). The complexes P123-PEI-R4/DNA and P123-PEI-R11/DNA were prepared according to 3.4 at the *w*/*w* radio of 5 and 2, respectively. A drop of the DNA/polymer polyplexes suspension was placed onto a copper grid. The excrescent solution was wiped off with lens wiping paper and the grid was dried at room temperature for several minutes before observation.

### 3.6. Agarose Gel Retardation Assay (Binging Capacity of Polymers/DNA Complexes)

Agarose gel electrophoresis was performed to investigate DNA binding ability of P123-PEI-R4 and P123-PEI-R11. Polymer/DNA complexes at different weight ratios ranging from 0.05 to 3.0 were prepared. These obtained complex solutions were analyzed by electrophoresis with the condition of 120 V and 30 min on a 1% (*w*/*v*) agarose gel in loading buffer. Then these gels were stained with 0.5 g/mL ethidium bromide for 15 min and illuminated by a UV illuminator to show the location of DNA.

### 3.7. Resistance to DNaseI Digestion and Serum

Stability of the polymer/DNA complexes was evaluated by testing the ability to protect plasmid DNA (pDNA) against DNaseI degradation. Generally, the designed amount of DNaseI solution were added to 10 µL complex solution (250 ng of pDNA) in 0.5 mL Eppendorf tubes, with the range of DNaseI doses per pDNA weight unit maintained between 0 and 10 U DNaseI/µg DNA, and incubated at 37 °C for 30 min. Then, 2 µL of 250 mM EDTA solution was added to each tube and incubated at room temperature for 10 min to inactivate DNaseI. Next, 10 µL 2 mg/mL sodium heparin was added to each tube and incubated at room temperature for 2 h to dissociate the complex completely. Stability of the complex to DNaseI digestion was further analyzed by electrophoresis.

In the experiments with serum, the complex solution (10 µL containing 10%, 25% and 50% serum) was obtained by adding different concentrations of FBS and incubating at 37 °C for 60 min. Then, electrophoresis was performed to determine the sensitivity of complex P123-PEI-R4 and P123-PEI-R11 to serum. In addition, the resistance of complex P123-PEI-R18 to DNaseI digestion and serum was used to compare with the sensitivity of complex P123-PEI-R4 and P123-PEI-R11.

### 3.8. Cytotoxicity Assay

The cytotoxicity of P123-PEI-R4 and P123-PEI-R11 polymer was measured by MTT assay. B16 cells were distributed to each well of a 96-well plate for 48 h. Previous medium was abandoned. 200 µL serum-free media with different concentrations of the polymer (0, 4, 8, 16, 24 and 32 µg/mL) were added to per cell well, respectively. After incubating the plate for 4 h with 5% CO_2_ at 37 °C, fresh growth medium was replaced and kept for 72 h. Thereafter, the wells were replaced with 20 µL 5 mg/mL sterilized MTT solution and 180 µL of fresh growth medium, and kept at 37 °C for 4 h. Subsequently, the MTT/growth medium was replaced by 150 µL DMSO and kept for 10 min with gentle vortexing at room temperature. The absorbance value at 570 nm was read by an ELISA plate reader with background subtraction. Cell viability was calculated using the following equation:

Cell viability (%) = (Absorbance of cells treated with nanoparticles − Absorbance of free medium alone)/(Absorbance of control untreated cells − Absorbance of free medium alone) × 100

### 3.9. In Vitro Gene Transfection

The transfection efficiency of P123-PEI-R4/DNA, P123-PEI-R11/DNA and P123-PEI-R18/DNA complexes in B16 cells was examined using the plasmid pEGFP-N2 and pGL3-Control. Cells were seeded in a 24-well plate at a density of 10^5^ cells per well in RPMI 1640 medium containing 10% FBS and incubated for 24 h until 80% confluence. Then, 100 µL polymer/DNA complex solution containing 2.5 µg DNA at various weight ratios (2–30) were added to the 24-well plate and incubated for 4 h at 37 °C in a 5% CO_2_ atmosphere. After that, the medium was replaced with 500 µL medium containing 10% FBS and then incubated for additional 48 h. The pEGFP-N2 expression ware was observed with an inverted fluorescent microscope (AE-31, Motic Corporation, Wetzlar, Germany).

The luciferase assay was conducted according to the manufacturer’s specifications. The medium was replaced with 100 µL cell culture lysis reagent (CCLR) and shaken for 30 min. After mixing with substrate, luciferase activity was examined with a luminometer (Turner Designs Luminometer Model TD-20/20, Promega Corp., Madison, AL, USA) as soon as possible. A bicinchoninic acid (BCA) protein assay was used to measure protein contents. Transfection efficiency for the pGL3-Control was calculated by the relative light units (RLUs) against the corresponding protein contents.

## 4. Conclusions

The new polyplexes P123-PEI-R4 and P123-PEI-R11 were successfully developed by cross-linking LMW PEI with P123 and then further coupled with peptide R4 and R11, respectively. Compared with branched PEI-25 kDa, the new polyplexes both showed suitable buffer capacity. In addition, these polyplexes could efficiently condense DNA into stable nanoparticles with proper sizes and zeta-potentials. Our gel retardation experiment confirmed PEI-P123-R4 had the ability to form complexes with DNA and was able to condense DNA effectively at a *w*/*w* ratio of 3.0. More importantly, P123-PEI-R11 was able to efficaciously condense DNA and neutralized its charge at a *w*/*w* ratio of 0.4, indicating that P123-PEI-R11 has a higher DNA binding ability. Furthermore, these polymers can effectively protect plasmid DNA from being degraded by DNaseI and have distinctly serum tolerance. Moreover, the new polyplex P123-PEI-R11 showed much lower cytotoxicity compared with P123-PEI-R4 and P123-PEI-R18 in B16 cell lines, it also exhibited the highest gene transfection efficiency compared with the other polyplexes (P123-PEI, P123-PEI-R4 and P123-PEI-R18) at any ratio. These results suggest that this new polymer (P123-PEI-R11) could be used as a potential safe and efficient non-viral carrier for future cancer gene therapy due to low cytotoxicity and high transfection.

## Abbreviations

The following abbreviations are used in this manuscript:

PEI	Polyethylenimine
P123	Pluronic P123
R4	RGD
R11	RGD-NLS
R18	RGD-TAT-NLS
LMW	Low molecular weight
HMW	High molecular weight
HLB	Hydrophile lipophilic balance
SMCC	*N*-succinimidyl-4-(*N*-maleimido-methyl) cyclohexane-1-carboxylate
MTT	3-(4,5-dimethyl-thiazol-2-yl)-2,5-diphenyl tetrazolium bromide
FBS	Fetal bovine serum
CCLR	Cell culture lysis reagent
BCA	Bicinchoninic acid
